# Benefits of Utilization of Hydroxy–Methionine in Diets of Finishing Pigs Raised Under Hot Environmental Conditions

**DOI:** 10.3390/vetsci13040397

**Published:** 2026-04-18

**Authors:** Caio Abércio da Silva, Cleandro Pazinato Dias, Marco Aurélio Callegari, Kelly Lais de Souza, José Henrique Barbi, Naiara Simarro Fagundes, Rafael Humberto de Carvalho

**Affiliations:** 1 Akei Animal Research, Estrada Vicinal Fartura–Areias, Km 3|Três Saltos, Fartura 18870-000, SP, Brazil; cleandropazinato@uol.com.br (C.P.D.); marco.callegari@akei.com.br (M.A.C.); kelly.souza@akei.com.br (K.L.d.S.); rafael.carvalho@uel.br (R.H.d.C.); 2 Adisseo France S.A.S., 10, Place du Général de Gaulle, 92160 Antony, France; jose.barbi@adisseo.com (J.H.B.); naiara.fagundes@adisseo.com (N.S.F.); 3 Animal Science Program, Center of Agrarian Science, State University of Londrina, Londrina 86057-970, PR, Brazil

**Keywords:** heat stress, methionine hydroxy analogue, performance, swine, meat quality

## Abstract

This study evaluated the effects of DL-methionine (DL-Met) and hydroxy–methionine (OH-Met) on the performance and meat quality of finishing pigs raised under intermittent hot environmental conditions. A total of 120 pigs (PIC337 × Camborough; 63.26 ± 4.49 kg initial body weight) were assigned to two dietary treatments in a randomized block design. Growth performance, carcass traits, and physicochemical meat parameters were assessed during the finishing period. Overall performance and carcass characteristics did not differ between treatments (*p* > 0.05). However, during finishing phase II (141–168 days), pigs fed OH-Met showed higher average daily gain (ADG; 1.027 vs. 0.957 kg/day; *p* < 0.05) and improved feed conversion ratio (FCR; 2.862 vs. 3.028; *p* < 0.05) compared with DL-Met. Meat from pigs receiving OH-Met presented a higher pH at 24 h postmortem (5.78 vs. 5.63; *p* = 0.022) and reduced water loss by pressure (26.14% vs. 28.78%; *p* = 0.047). No differences were detected for backfat thickness, longissimus dorsi depth, lean percentage, color parameters, or lipid oxidation (TBARS) (*p* > 0.05). In conclusion, under intermittent heat stress conditions during the late-finishing phase, diets formulated with OH-Met resulted in improved growth efficiency and reduced meat water loss compared with DL-Met diets.

## Simple Summary

Methionine is an essential sulfur-containing amino acid and is one of the main nutrients limiting growth in pigs. The most widely used synthetic sources in swine diets are DL-methionine (DL-Met) and hydroxy–methionine (OH-Met). Because high environmental temperatures can impair feed efficiency and meat quality, selecting the most effective methionine source under warm conditions is important for pig production. This study compared DL-Met and OH-Met in finishing pigs raised in a warm climate. Both sources supported similar overall growth performance and carcass characteristics. However, during the final stage of finishing, when pigs were heavier and more susceptible to heat stress, animals fed OH-Met showed improved feed efficiency and reduced water loss in the meat. These results indicate that although both methionine sources are effective, OH-Met may provide advantages during late finishing under hot conditions. Therefore, OH-Met can be considered a strategic nutritional option for growing–finishing systems located in warm regions.

## 1. Introduction

Methionine is a sulfur-containing amino acid (SAA) considered limiting for pigs, ranking behind lysine and threonine [1], and it is commonly supplemented in pig diets in synthetic form [2]. Methionine participates in several functions in different biological processes. For protein deposition, it acts as a sulfur donor to generate two other SAAs: cysteine and cystine [3]. Cysteine, in turn, is a component of other molecules, such as glutathione and taurine, which are important for maintaining the redox balance of animals by acting against oxidative stress [4,5,6]. Additionally, methionine is an important donor of methyl groups for other molecules involved in cell proliferation and differentiation [7]. Methionine is the major driver of DNA methylation, as it is essential for the synthesis of S-adenosylmethionine [8].

The most commonly used synthetic sources of methionine are DL-methionine (DL-Met), L-methionine (L-Met), and DL-2-hydroxy-4-methylthiobutanoic acid, also known as hydroxy–methionine (OH-Met). The bioavailability of these sources is generally considered similar [2,9] when diets are supplemented with equimolar levels of the amino acid [10,11,12]. Nevertheless, Schermuly et al. [13] found that pigs supplemented with OH-Met showed alterations in secondary bile acid metabolites in the jejunum and sphingosine metabolites in the ileum, respectively, suggesting improved digestive and immune efficiency of OH-Met when compared to DL-Met and L-Met.

Although positive responses to both methionine sources have been reported in pigs under heat stress conditions, comparative evaluations under hot environments have been more extensively studied in broilers [14,15,16]. These studies showed that OH-Met supplementation partially mitigated the growth-depressing effects of chronic heat stress compared with DL-Met and improved antioxidant status. This effect was associated with higher hepatic ratios of reduced glutathione (GSH) to total GSH in animals supplemented with OH-Met.

In growing–finishing pigs, methionine supplementation above the requirement (20% above requirement) improved performance under hot conditions, regardless of the source used; however, there were indications favoring OH-Met over DL-Met for certain quantitative carcass traits [12]. These authors reported greater lumbar muscle depth in pigs fed OH-Met during the growing and finishing phases under hot conditions. However, the study was conducted in lighter pigs (approximately 106 kg live weight), which are generally less susceptible to heat stress [17], a condition attributed, among other factors, to increased metabolic heat production and a reduced surface area-to-mass ratio, making these animals less efficient at dissipating heat through this important pathway [18]. The present study aimed to further evaluate the effects of DL-Met (99%) and OH-Met (88%) on the performance, carcass characteristics, and meat quality of pigs raised under intermittent hot environmental conditions, a common scenario in many tropical countries where swine production represents an important economic activity, during the finishing phase and slaughtered at high live weight, a trend increasingly observed in commercial production systems worldwide.

## 2. Materials and Methods

The experiment was conducted according to the guidelines of the Brazilian National Council for the Control of Animal Experimentation (CONCEA) and approved by the Ethics Committee for Animal Experiments of Akei Animal Research (protocol no. 016/19).

### 2.1. Animals and Housing

A total of 120 crossbred pigs (PIC337 × Camborough), comprising 60 surgically castrated males and 60 gilts, with an initial body weight of 63.26 ± 4.49 kg and 105 days of age, were used. Animals were allocated to 24 pens (5 pigs per pen), resulting in 12 pens per treatment. Each pen (5.5 m^2^) was equipped with a nipple drinker and a single-space Dutch feeder (35 cm width). Thermal control was performed manually by operating curtains on the sides of the barn. Ambient temperature and relative humidity were recorded every 30 s using a data logger (Instrutemp ITLOG 80, São Paulo, Brazil) positioned at pig shoulder height (70 cm) in the center of the barn. The Temperature–Humidity Index (THI) was calculated and classified as <75 (no heat stress), 75–78 (moderate), 79–83 (severe), and ≥84 (extremely severe) [19].

The Temperature–Humidity Index (THI) was calculated according to the National Research Council [20] using the following equation:

THI = (1.8 × T + 32) − [(0.55 − 0.0055 × RH) × (1.8 × T − 26)]

where T is ambient temperature (°C) and RH is relative humidity (%).

Environmental monitoring demonstrated cyclic daily oscillations in THI values, characterizing intermittent rather than constant heat stress exposure across both finishing phases.

### 2.2. Experimental Treatments and Diets

A randomized complete block design was used. Blocks were defined based on initial body weight and sex, and within each block, pens were randomly assigned to one of the two methionine sources, DL-Met (99% of active ingredient, powder form) or OH-Met (88% of active ingredient, liquid form), on an equimolar basis. The experimental diets were prepared based on corn and soybean meals and formulated to meet the minimum nutritional requirements for pigs according to Rostagno et al. [21] (Table 1). The experimental diets were offered during finishing phase I, from 105 to 140 days of age, and finishing phase II, from 141 to 168 days of age. The animals were sent to slaughter with an average weight of 126.81 ± 5.48 kg. Feed and water were provided ad libitum throughout the experimental period.

### 2.3. Performance and Carcass Trait Analyses

Pigs were weighed individually, and feed intake was recorded per pen at 105, 140, and 168 days of age. These values were used to calculate the individual body weight (BW), average pen daily weight gain (ADG), average pen daily feed intake (ADFI), and average pen feed conversion ratio (FCR).

At 168 days of age, all animals were sent for slaughtering. Feed was withdrawn 12 h before transportation, while water remained available until slaughtering. In accordance with current legislation, animals were electrically stunned using a head-only stunner (Petrovina IS-2000, Brasfood Equipamentos Frigoríficos, Sertãozinho, SP, Brazil) applying 350 V and 1.3 A for approximately 3 s, followed by exsanguination by severing the major blood vessels of the neck.

After slaughtering, scalding, and evisceration, the carcasses were split longitudinally and refrigerated at 2 ± 1 °C for 24 h in a cooling chamber. Each carcass was weighed and subjected to electronic grading (using the Hennessy Grade Probe, Hennessy Grading Systems, Auckland, New Zealand), which determined the backfat thickness (mm), loin depth (mm) measured at point P2, and lean meat weight (kg) in the carcass.

### 2.4. Meat Quality Assessments

Twenty carcasses per treatment were selected for meat quality evaluation, balancing sex (barrows and females) within the block. At 24 h postmortem, carcasses were evaluated in the cutting room (4 °C). Muscle pH was measured using a portable pH meter equipped with an integrated temperature probe (Testo 205, Testo AG, Lenzkirch, Germany). The instrument was calibrated at room temperature with standard buffer solutions (pH 4.0 and 7.0) prior to the measurements. Readings were obtained in the left Longissimus thoracis muscle (between the last and penultimate ribs) and in the proximal portion of the left Semimembranosus muscle, with a single measurement taken per sample. The electrode was rinsed with distilled water between measurements to prevent cross-contamination. At the time of pH determination, the mean internal temperatures were 0.9 °C for the Longissimus thoracis and 2.8 °C for the Semimembranosus.

Color measurements were performed after a total blooming time of 30 min. A portable colorimeter (CR-10, Konica Minolta, Osaka, Japan) was used with illuminant D65, a 10° observer angle, and an 8 mm aperture. Three readings were taken per sample and averaged. Results are expressed in the CIELAB system (L*, a*, b*).

Marbling score and shear force (SF, kgf) were also determined according to established methodologies [22]. In the *Longissimus dorsi* muscle, the following parameters were evaluated at 24 h, 120 h, and 10 days postmortem: pH, water loss by pressure (LWP) [23], instrumental color (L*, a*, b*), and lipid oxidation measured as thiobarbituric acid reactive substances (TBARS), expressed as mg malondialdehyde (MDA)/kg of meat [24,25].

### 2.5. Statistical Analysis

Performance data were analyzed in R (version 3.5.1; R Foundation for Statistical Computing, Vienna, Austria) using linear mixed-effects models under a randomized complete block design. The pen was the experimental unit for growth performance traits (ADFI, ADG, and FCR; 12 pens per treatment). Models included methionine source (DL-Met vs. OH-Met), sex, phase (when applicable), and their interactions as fixed effects. Block was included as a random effect, and pen was modeled as a random term nested within block and treatment to properly account for the hierarchical structure of the data.

For individual body weight, carcass traits, and meat quality variables measured once, the individual pig was treated as the observational unit while accounting for clustering within pens. Accordingly, pen (nested within block and treatment) was included as a random effect to avoid pseudo-replication. Fixed effects included methionine source, sex, and their interaction.

Meat quality traits evaluated at 24 h, 120 h, and 10 d postmortem were analyzed using repeated-measures mixed models including methionine source, sex, time, and their interactions as fixed effects. Block and pen were included as random effects, and pig was specified as the repeated subject within the pen. The residual covariance structure for repeated measures was selected based on the lowest Akaike Information Criterion (AIC) among competing structures and is reported for each endpoint.

Residual normality and homoscedasticity were assessed using Shapiro–Wilk and Levene’s tests, respectively, complemented by visual inspection of diagnostic plots. Pairwise comparisons were performed using Tukey-adjusted tests. Statistical significance was declared at *p* ≤ 0.05, and 0.05 < *p* ≤ 0.10 was considered indicative of a trend.

## 3. Results

**Figure 1:**
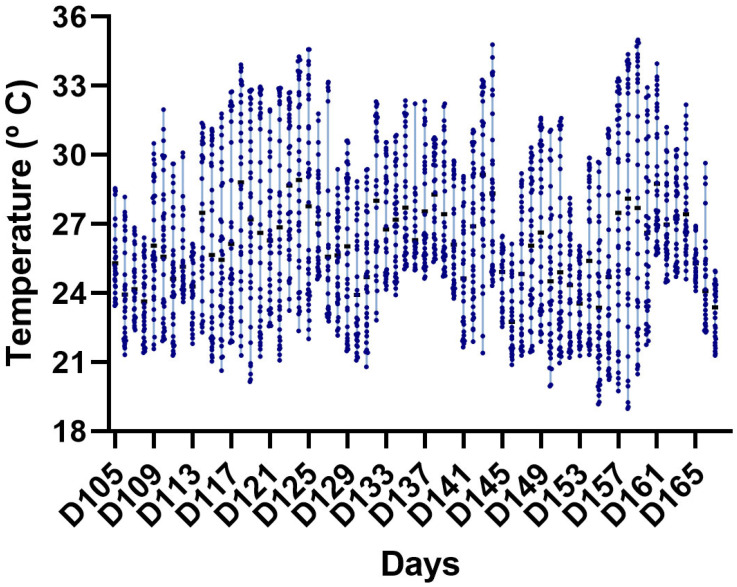
Ambient temperature recorded inside the experimental facility during the growing–finishing period. Data are presented as daily mean values, with vertical bars indicating the minimum and maximum recorded values. The dots represent individual measurements, and the cubes represent the daily mean values.

**Figure 2:**
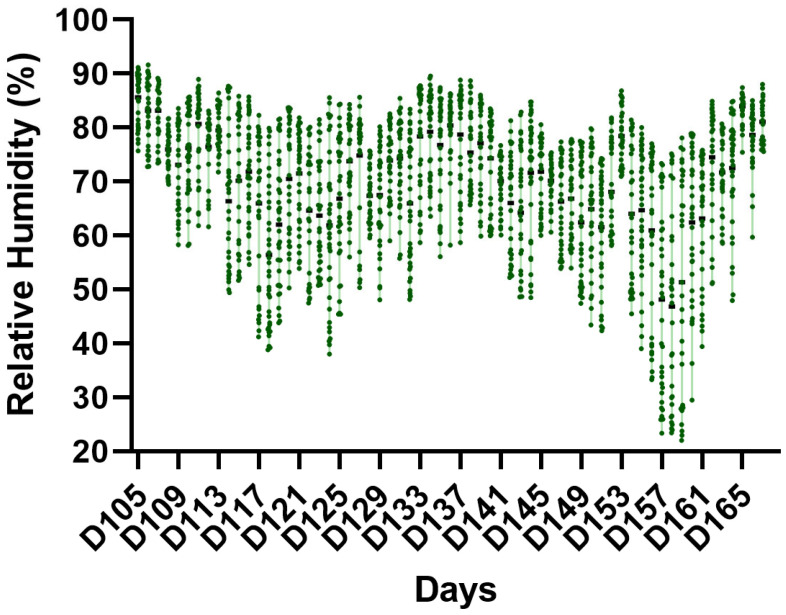
Relative humidity recorded inside the experimental facility during the growing–finishing period. Data are presented as daily mean values, with vertical bars indicating the minimum and maximum recorded values.

**Figure 3:**
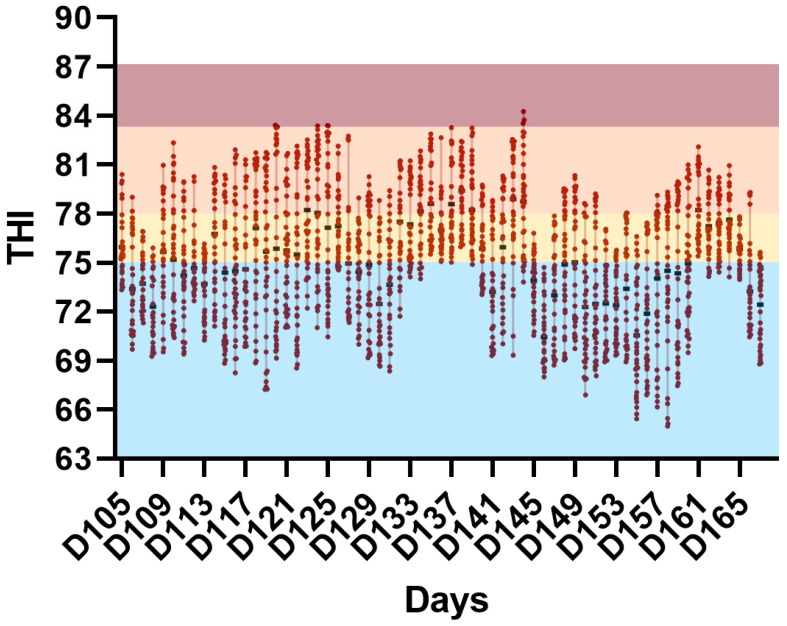
Temperature–Humidity Index (THI) recorded inside the experimental facility during the growing–finishing period. THI values were calculated based on ambient temperature and relative humidity data and are presented as daily mean values, with vertical bars indicating the minimum and maximum recorded values. THI values were classified according to heat stress categories using color coding as follows: blue (<75) = absence of heat stress; yellow (75–78) = moderate heat stress; orange (79–83) = severe heat stress; red (≥84) = extremely severe heat stress.

The average minimum and maximum temperatures (Figure 1) and relative humidity (Figure 2) during the experimental period were 21.16 ± 1.20 °C and 29.71 ± 2.44 °C, respectively, and 66.92 ± 14.91%, with lower and upper temperature limits of 18.98 and 35.00 °C, respectively. Considering the two experimental phases (finishing phases I and II), the percentage of days with temperatures above 21 °C did not differ between the periods (*p* > 0.05). Mean daily THI, maximum daily THI, minimum daily THI, and the percentage of hours/day with THI ≥ 75 did not differ between finishing phases I and II (*p* > 0.05), confirming a similar pattern of thermal challenge across phases. When evaluating the THI, it was observed that the animals spent approximately 46.19% of the experimental period under no heat stress conditions and 34.75% and 19.16% under moderate and severe heat stress conditions, respectively. The THI values observed during the study (Figure 3) indicate intermittent thermal stress conditions, with pronounced fluctuations even within a 24 h period, frequently oscillating between the absence of thermal stress and severe heat stress. The environmental conditions indicate exposure to intermittent heat stress patterns, characterized by daily cyclic variation in THI values rather than continuous exposure to a high thermal load.

### 3.1. Growth Performance

No significant effects of methionine source were observed during finishing phase I (105–140 d) for ADFI (*p* = 0.823), ADG (*p* = 0.741), or FCR (Table 2; *p* = 0.705). The average daily feed intake was 2.613 and 2.602 kg/d, ADG was 1.033 and 1.023 kg/d, and FCR was 2.530 and 2.550 for DL-Met and OH-Met, respectively. In contrast, during finishing phase II (141–168 d), the methionine source affected ADG (*p* = 0.017) and FCR (*p* = 0.013). Pigs fed OH-Met exhibited greater ADG compared with those fed DL-Met (1.027 vs. 0.957 kg/d), representing an absolute increase of 0.070 kg/d and a relative improvement of 7.3%. Similarly, FCR was improved in pigs receiving OH-Met (2.862 vs. 3.028), corresponding to an absolute reduction of 0.166 units and a 5.5% enhancement in feed efficiency. The average daily feed intake did not differ between treatments in this phase (2.934 vs. 2.891 kg/d; *p* = 0.498), indicating that the performance improvement was associated with enhanced feed efficiency rather than increased feed consumption.

When the entire experimental period (105–168 d) was considered, no significant differences were detected for ADFI (*p* = 0.781), ADG (*p* = 0.252), or FCR (*p* = 0.197).

**Table 2:** Effects of DL-methionine and hydroxy–methionine supplementation on growth performance and body weight of finishing pigs during distinct finishing phases.

	Finishing Phase I (105–140 d)			Finishing Phase II (141–168 d)			Total (105–168 d)			Individual BW, kg		
	ADFI, kg/d	ADG, kg/d	FCR	ADFI, kg/d	ADG, kg/d	FCR	ADFI, kg/d	ADG, kg/d	FCR	105 d	141 d	168 d
DL-Met	2.613	1.033	2.530	2.891	0.957 ^b^	3.028 ^a^	2.737	0.999	2.737	63.27	99.44	126.31
OH-Met	2.602	1.023	2.550	2.934	1.027 ^a^	2.862 ^b^	2.750	1.025	2.686	63.26	99.06	128.02
SEM	0.043	0.015	0.034	0.046	0.014	0.052	0.042	0.010	0.036	0.929	1.101	1.082
*p*-value	0.823	0.741	0.705	0.498	0.017	0.013	0.781	0.252	0.197	0.981	0.705	0.144

DL-Met = DL-methionine; OH-Met = hydroxy–methionine. Values are expressed as least squares means. SEM = standard error of the mean. ^a,b^ Different lowercase letters within the same column indicate significant differences (*p* ≤ 0.05). ADFI = average daily feed intake; ADG = average daily gain; FCR = feed conversion ratio; BW = body weight.

### 3.2. Carcass and Meat Quality

Methionine source did not affect carcass characteristics or physical meat quality parameters (Table 3). Final carcass weight was similar between treatments (90.66 vs. 91.18 kg for DL-Met and OH-Met, respectively; *p* = 0.479), corresponding to a non-significant absolute difference of 0.52 kg (0.6%). Backfat thickness (16.42 vs. 16.03 mm; *p* = 0.530) and loin depth (60.50 vs. 60.65 mm; *p* = 0.919) were not influenced by dietary treatment, with minimal numerical variation between groups. Lean meat weight also did not differ (49.73 vs. 50.30 kg; *p* = 0.288), representing a non-significant increase of 0.57 kg (1.1%) in pigs fed OH-Met. Marbling scores were comparable between treatments (1.954 vs. 2.132; *p* = 0.445). Similarly, shear force values were also similar (5.246 vs. 5.069 kgf; *p* = 0.620), indicating no impact on meat tenderness.

At 24 h postmortem, pigs supplemented with OH-Met exhibited a higher pH compared with DL-Met (5.778 vs. 5.632; +0.146 units, corresponding to a 2.6% increase; *p* ≤ 0.05). However, no differences between treatments were detected at 120 h or 10 days postmortem (*p* > 0.05). Across time, pH values increased from 24 h to 120 h and remained stable thereafter (Table 4; *p* = 0.002). For lightness (L), redness (a), and yellowness (b), no significant effects of treatment, time, or interaction were detected (*p* > 0.05). A significant treatment × time interaction was also observed for water loss by pressure (LWP) (*p* = 0.047). At 120 h postmortem, pigs receiving OH-Met showed lower LWP compared with DL-Met (26.14% vs. 28.78%; −2.64 percentage points, representing a 9.2% reduction; *p* ≤ 0.05). No treatment differences were detected at 24 h or 10 days postmortem (*p* > 0.05). Finally, lipid oxidation assessed by TBARS (mg MDA/kg) was not influenced by treatment, time, or their interaction (*p* > 0.05).

**Table 4:** Effects of methionine source and postmortem time on meat quality traits in finishing pigs.

Parameter	Time	Treatments		*p*-Value		
		DL-Met	OH-Met	Treatment	Time	Treat × Time
pH	24 h	5.632 ^b^ ± 0.032	5.78 ^a^ ± 0.032	0.012	0.002	0.001
	120 h	5.792 ± 0.026	5.842 ± 0.026	—	—	—
	10 d	5.820 ± 0.023	5.855 ± 0.023	—	—	—
L*	24 h	49.00 ± 0.452	48.05 ± 0.452	0.804	0.699	0.785
	120 h	49.56 ± 0.462	48.42 ± 0.462	—	—	—
	10 d	49.089 ± 0.594	47.490 ± 0.594	—	—	—
a*	24 h	3.269 ± 0.168	3.160 ± 0.168	0.781	0.398	0.870
	120 h	4.563 ± 0.224	4.387 ± 0.224	—	—	—
	10 d	3.687 ± 0.173	3.270 ± 0.173	—	—	—
b*	24 h	11.23 ± 0.166	11.07 ± 0.166	0.789	0.841	0.881
	120 h	13.12 ± 0.214	12.49 ± 0.214	—	—	—
	10 d	12.556 ± 0.174	12.194 ± 0.174	—	—	—
Water loss by pressure (%)	24 h	29.60 ± 0.661	28.54 ± 0.661	0.156	0.105	0.047
	120 h	28.78 ^a^ ± 0.673	26.14 ^b^ ± 0.673	—	—	—
	10 d	27.610 ± 0.449	28.268 ± 0.449	—	—	—
TBARS (mg MDA/kg)	24 h	0.095 ± 0.002	0.090 ± 0.002	0.205	0.102	0.278
	120 h	0.090 ± 0.002	0.087 ± 0.002	—	—	—
	10 d	0.106 ± 0.003	0.110 ± 0.003	—	—	—

DL-Met = DL-methionine; OH-Met = hydroxy–methionine; SEM = standard error of the mean. Values are least squares means ± SEM obtained from a linear mixed model including treatment, time, and treatment × time interaction as fixed effects, with animal as a random effect and time as a repeated measure within animal (covariance structure selected by information criteria, e.g., AIC). ^a,b^ Different lowercase letters within the same time point indicate differences between treatments (Tukey- or Sidak-adjusted simple effects, *p* ≤ 0.05).

## 4. Discussion

The THI values recorded throughout the experimental period (Figure 3) confirm that heat stress conditions were intermittent, with pronounced fluctuations throughout the day. Thus, in addition to the direct effects of THI values above 75, which represent moderate to extremely severe heat stress at different time points during the study, the high daily amplitude of THI should also be considered, particularly because it may disrupt circadian rhythm, reduce feed intake, and consequently impair performance [26].

The results indicate that both synthetic methionine sources supplemented at equimolar levels were effective in supporting the overall growth performance of finishing pigs under the thermal conditions evaluated. Specifically, in finishing phase I, no differences were observed between treatments for any performance parameter. However, positive effects on ADG and FCR were observed in finishing phase II with OH-Met supplementation, without resulting in an overall advantage for either treatment when both phases were considered together.

The frequency of ambient temperatures above 21 °C in finishing phases I and II did not differ between periods, indicating that the impact of intermittent heat stress on performance in finishing phase I was not influenced by the methionine source evaluated. In contrast, during finishing phase II, when animals are heavier, and metabolic heat production is greater, conditions that may amplify subtle differences in nutrient utilization efficiency and consequently performance, OH-Met supplementation may have contributed to the observed improvement [27,28].

Although animals were evaluated throughout the finishing period, the body weight range in finishing phase I compared with finishing phase II implies different thermal comfort requirements. For the body weight ranges evaluated in the present study, Payola and Piriou [29] indicate that heavier animals (represented here by pigs in finishing phase II) require an average comfort temperature approximately 2 to 3 °C lower than animals within the body weight range corresponding to finishing phase I.

These differences can be explained by the fact that heavier pigs generally present a greater proportion of adipose tissue relative to other tissues [30], which represents an important limiting factor for heat dissipation [31], thereby predisposing animals to a greater sensation of thermal stress. Furthermore, as body weight increases, metabolic heat production also increases, while the surface area-to-mass ratio decreases, reducing the efficiency of heat loss through this important thermoregulatory pathway [18]. Under these conditions, oxidative stress risk and maintenance energy requirements increase, which may elevate the importance of sulfur amino acids involved in antioxidant metabolism and protein turnover. Consequently, differences in metabolic utilization between methionine sources may become more evident in heavier animals exposed to heat stress conditions [4,5,18,32].

Even under intermittent thermal stress conditions, in which animals experience heat stress, intestinal absorption capacity may change [27,28]. Absorption may shift from carrier-dependent to carrier-independent pathways, whereas diffusion remains unaffected. DL-Met is absorbed by active transporters present throughout the intestine [33]. On the other hand, OH-Met, due to its characteristics as an organic acid, is absorbed mainly before reaching the intestine by passive diffusion, with absorption occurring predominantly in the upper gastrointestinal tract [34,35]. Therefore, based on previous studies, OH-Met, due to earlier absorption in the digestive tract, undergoes less action from the intestinal microflora, resulting in potentially improved utilization when compared to DL-Met [34,35,36]. These findings may help explain the results observed in the present study.

Under heat stress, intestinal permeability and transporter activity may be altered, potentially compromising active amino acid transport [27,28]. In this context, the passive diffusion characteristics of OH-Met may confer greater stability of absorption, maintaining methionine availability when carrier-mediated mechanisms are partially impaired [37,38]. The passive absorption of OH-Met probably leads to an increased availability of methionine for extraintestinal tissues and may contribute to the availability of other amino acids, such as lysine, because of reduced competition at the intestinal site of active amino acid absorption [39]. This mechanism may be particularly relevant during late finishing, when feed intake patterns fluctuate under heat stress, and efficient post-absorptive amino acid utilization becomes crucial for sustaining the growth rate [12,27].

OH-Met has a potentially greater role than DL-Met in the formation of L-cysteine, which in turn participates in protein synthesis and is a precursor of glutathione or is catabolized into taurine [36,40], two compounds with antioxidant functions [41,42]. Based on these metabolic relationships, it has been suggested that dietary OH-Met supplementation may increase circulating taurine concentrations; however, plasma taurine levels were not measured in the present study. Additionally, blood flow and net absorption of amino acids in the portal vein of pigs have been reported to improve upon dietary supplementation with OH-Met, resulting in increased zootechnical performance [43,44]. Zhang et al. [39], working with sows, observed higher plasma concentrations of leucine, isoleucine, and valine in animals supplemented with OH-Met compared with those receiving DL-Met.

Furthermore, in swine, dietary supplementation with OH-Met has been associated with alterations in secondary bile acid metabolites in the jejunum and sphingosine metabolites in the ileum, suggesting improved digestive and immunological efficiency of OH-Met compared with DL-Met and L-Met [13], attributes that may favor improved nutrient utilization.

Collectively, these factors may contribute to greater ADG and improved FCR observed with OH-Met supplementation during the late finishing phase in pigs raised under hot conditions (finishing phase II). Additionally, as an organic acid with a pKa of 3.53, OH-Met may modulate the intestinal microbiota and improve intestinal health status, potentially enhancing nutrient absorption [45].

Particularly regarding the positive effects on performance observed only in the final finishing phase, it is important to highlight that, at this stage, animals exhibit the highest feed intake. Therefore, optimizing feed efficiency at this point, particularly through improvements in feed conversion, has relevant economic implications under commercial production conditions. Information regarding the influence of methionine source on the performance of growing–finishing pigs under heat stress conditions remains limited [12]. Although acknowledging differences between species and production categories, in broilers subjected to heat stress, dietary supplementation with OH-Met resulted in improved performance compared to birds supplemented with DL-Met [16]. Furthermore, under intermittent heat stress conditions in broilers during the final finishing phase (21 to 42 days of age), a scenario comparable to the present study, improved feed conversion was also observed in birds fed diets formulated with OH-Met compared with DL-Met [46].

Methionine is a precursor of cysteine, which participates in the synthesis of glutathione and taurine, important metabolic antioxidants. Although no differences in TBARS were detected in the present study, the enhanced potential of OH-Met to support glutathione and taurine synthesis may contribute to improved cellular redox balance without necessarily resulting in measurable differences in lipid peroxidation under the specific storage conditions evaluated [47,48]. Oxidative damage is increased under heat stress conditions [49], and this condition may intensify oxidative challenges in muscle tissue [50].

Based on the findings of these studies, the results observed in the current study, only in the last phase of fattening, can be explained by the fact that heavier animals are more susceptible to the adversities of heat stress [51], a condition associated with greater metabolic changes and oxidative stress [52,53].

In general, it was observed that the animals that received OH-Met did not differ from those supplemented with DL-Met for carcass characteristics; however, the use of OH-Met was associated with some improvements in meat quality parameters. The results obtained for carcass characteristics are in line with the findings of Da Silva et al. [12], which indicate that different sources of methionine have little impact on these parameters, except under conditions in which the doses of this amino acid are increased in the diet.

Temperature limits of the thermal comfort zone for finishing pigs have been established to be between 17 and 21 °C. In the present study, the temperature record indicates that the animals underwent periods of heat stress throughout the experiment, which may have favored the positive responses of dietary supplementation with OH-Met in some variables related to meat quality, such as pH at 24 h and water loss by pressure measured at 120 h postmortem. The delayed effect observed at 120 h postmortem may reflect cumulative structural changes in muscle proteins, where small differences in oxidative stability and pH decline dynamics become more evident during extended storage rather than immediately after slaughter [28,47].

One of the most important variables for meat quality is pH, which shows a positive correlation with water retention capacity, color parameter *a and tenderness, and a negative correlation with *L and meat water loss [54,55]. In the postmortem period, muscle glycogen undergoes glycolysis and is converted into lactic acid [56], which reduces muscle pH. However, a pH lower than the ideal level reduces both the ability of muscle proteins to bind water and the negative electrostatic repulsion between thick (myosin) and thin (actin) filaments, shortening the spaces between them, causing shrinkage of the myofibrils [57], and consequently impairing the water retention capacity and tenderness of the meat.

The drop in muscle pH after slaughter is therefore expected, and it is affected by the temperature at which animals are raised [58]. Under the heat stress conditions experienced by the animals in the current study, the production of oxygen-reactive substances and the risk of oxidative stress are likely to damage cell walls and tissues, increasing water loss from the meat [50,52,59,60]. The higher pH observed in the muscle of animals supplemented with OH-Met may be associated with the antioxidant action exerted by this source of methionine [49], which is capable of alleviating the response to heat stress [39] and is reported to be more effective as a precursor of glutathione and taurine, important antioxidants. Because early postmortem pH decline influences subsequent protein denaturation and the spatial distribution of water within the myofibrillar matrix, the higher pH observed at 24 h may have contributed to the improved water-holding capacity detected at 120 h postmortem [28,61]. Preventing protein oxidation reduces the biological changes that these molecules undergo [62], minimizing damage related to water retention capacity and its consequences [63].

Our findings are supported by studies that have demonstrated the link between impaired water retention in meat and heat stress conditions associated with oxidative damage [39]. These observations are further supported by evidence indicating a greater potential of OH-Met to support the synthesis of glutathione and taurine compared with DL-methionine [15]. Yang et al. [64] found that meat from pigs exposed to prolonged periods of heat stress presented higher values of water loss by dripping and shear force measured in the *Longissimus* muscle. Similarly, Liu et al. [59] observed that chronic heat stress (33 ± 2 °C) resulted in a higher incidence of PSE (pale, soft, and exudative) meat in growing pigs, a condition characterized by greater water loss. This was also demonstrated by an increase in L* and b* values and a reduction in a* during 45 min to 96 h postmortem, along with a significant reduction in muscle glycogen content and an increase in water loss during cooking. In addition, chronic heat stress determined an increase in the malondialdehyde content in the muscle and blood serum and promoted a reduction in enzymes related to the antioxidant system, such as glutathione peroxidase (GSH-Px) in the serum.

In the present trial, no effects of methionine sources on TBARS were observed, which is consistent with the results obtained by Rasch et al. [48], who worked with nursery piglets fed diets balanced with three sources of methionine (L-Met, DL-Met, and OH-Met). These authors did not observe differences in plasma oxidation parameters but found a greater abundance of genes linked to both GSH synthesis and oxidative stress status in pigs supplemented with OH-Met compared to the other synthetic methionine sources, supporting the potential of OH-Met as a methionine source to minimize oxidative damage. Therefore, the absence of differences in TBARS should not be interpreted as a lack of metabolic effect but rather as an indication that oxidative modulation by methionine source may occur at molecular or enzymatic levels not fully captured by lipid peroxidation assays alone [48,65].

## 5. Conclusions

This study further demonstrated that OH-Met and DL-Met are comparable sources of synthetic methionine for finishing pigs. However, when compared during the final phase of the finishing period and under cyclic hot environmental conditions, OH-Met was associated with greater weight gain, improved feed conversion, and reduced meat water loss. These findings suggest that OH-Met may offer advantages for growing–finishing systems located in regions with intermittent hot climatic conditions, with potential positive economic implications, considering that improved feed efficiency was observed during the phase in which pigs exhibit the highest feed intake.

## Figures and Tables

**Table 1:** Composition and nutritional values of experimental diets for finishing phase I (105–140 days of age) and finishing phase II (141–168 days of age).

Ingredients, % of Feed	Finishing Phase I	Finishing Phase II
	DL-Met/OH-Met	DL-Met/OH-Met
Corn	80.466	84.188
Soybean meal	14.855	11.584
Soybean oil	1.401	1.076
Dicalcium phosphate	1.101	1.049
Limestone	0.588	0.575
L-lysine	0.435	0.439
Salt	0.341	0.329
Vitamin premix ^1^	0.100	0.100
Mineral premix ^2^	0.100	0.100
Selenium–Methionine ^3^	0.045	0.045
Synthetic methionine source ^4,5^	0.097/0.108	0.061/0.069
L-threonine	0.138	0.132
L-tryptophan	0.055	0.058
L-valine	0.043	0.044
Antioxidant	0.010	0.010
Zn Bacitracin	0.015	-
Adsorbent	0.200	0.200
Nutrients, %		
Metabolizable energy, kcal/kg	3350	3350
Crude protein	14.00	12.82
Crude fiber	2.360	2.209
Lysine SID	0.882	0.811
Lysine total	0.971	0.894
Met +Cist SID	0.443	0.379
Met + Cys total	0.494	0.428
Methionine SID	0.290	0.243
Methionine total	0.313	0.265
Threonine SID	0.573	0.527
Threonine total	0.626	0.573
Tryptophan SID	0.176	0.162
Tryptophan total	0.196	0.180
Valine SID	0.609	0.560
Valine total	0.692	0.636
Available phosphorus	0.283	0.269
Total phosphorus	0.528	0.509
Calcium	0.573	0.545
Chloride	0.259	0.252
Sodium	0.165	0.158
Potassium	0.389	0.365

^1^ Vitamin premix provided 6000 IU vitamin A, 1500 IU vitamin D3, 15 mg vitamin E, 1.5 mg vitamin K3, 1.35 mg vitamin B1, 4 mg vitamin B2, 2 mg vitamin B6, 20 µg vitamin B12, 20 mg niacin, 9.35 mg pantothenic acid, 600 µg folic acid, and 80 mg biotin per kg of diet. ^2^ Mineral premix provided 100 mg Fe, 10 mg Cu, 40 mg Mn, 1 mg Co, 100 mg Zn, and 1.5 mg I per kg of diet. ^3^ Selisseo 0.2%. Standardized ileal digestible (SID) amino acids calculated according to Rostagno et al. [21]. ^4^ DL-Methionine (99% methionine total and SID; and 99% Met + Cys total and SID). ^5^ OH-Methionine (88% methionine total and SID; and 88% Met + Cys total and SID).

**Table 3:** Effects of DL-methionine and hydroxy–methionine supplementation on carcass characteristics and physical meat quality traits in finishing pigs.

	Carcass, kg	BT, mm	LD, mm	LM, kg	MAR	SF, kgf
DL-Met	90.66	16.42	60.50	49.73	1.954	5.246
OH-Met	91.18	16.03	60.65	50.30	2.132	5.069
SEM	0.477	0.314	0.760	0.290	0.113	0.171
*p*-value	0.479	0.530	0.919	0.288	0.445	0.620

DL-Met = DL-methionine; OH-Met = hydroxy–methionine. Values are presented as least squares means. SEM = standard error of the mean. BT = backfat thickness; LD = loin depth; LM = lean meat weight in the carcass; MAR = marbling score; SF = shear force. No significant differences were detected between treatments (*p* > 0.05).

## Data Availability

The original contributions presented in this study are included in the article. Further inquiries can be directed to the corresponding author.
